# Chemical and Flavor Characteristics of Enzyme-Modified Cheese Made by Two-Stage Processing

**DOI:** 10.3390/gels8030160

**Published:** 2022-03-04

**Authors:** Peng Gao, Yanling Su, Wenyuan Zhang, Xiaoyang Pang, Ning Xie, Min Zhang, Jiaping Lv, Shuwen Zhang

**Affiliations:** 1 Beijing Advanced Innovation Center for Food Nutrition and Human Health, Beijing Technology & Business University, Beijing 100048, China; 82101202182@caas.cn; 2 Institute of Food Science and Technology, Chinese Academy of Agricultural Sciences, Beijing 100193, China; 82101195218@caas.cn (Y.S.); zhangwenyuan@caas.cn (W.Z.); pangxiaoyang@caas.cn (X.P.); xiening@caas.cn (N.X.)

**Keywords:** enzyme-modified cheese, proteolysis, lipolysis, flavor compounds, aroma compounds

## Abstract

(1) Background: to date, a clear description of the impact of specific enzymes on the enzyme-modified cheese (EMC) flavor is lacking. Moreover, comparative studies on the aroma compounds’ intensity of EMC have been rarely investigated. Therefore, this study was done to determine the influence of incubating substrates with proteases and different lipases on cheese ripening index and aroma compounds. (2) Methods: two-stage processing was adopted; proteolysis followed by lipolysis. (3) Results: results showed that the usage of Flavourzyme may improve the value of pH 4.6-WSN/TN%. Butanoic acid and hexanoic acid have a significant influence on the overall flavor of EMCs. In particular, the ethenyl acetate compound was detected in all products and was perceived as a fruity and sweet aroma, which has not been reported in previous literature. The concentration of short-chain fatty acids of EMCs made by Lipase MER was higher than EMCs made by Palatase, while the total content of medium and long-chain fatty acids of EMCs made by Lipase MER was lower than EMCs made by Palatase. The percentage of esters compounds in EMCs made by Lipase AY 30G was higher than the other two lipases, except EMC1. (4) Conclusions: Flavourzyme may be used to speed up the ripening of cheeses that need extensive proteolysis. The ability of Lipase MER to hydrolyze short-chain fatty acids was stronger than that of Palatase, while the ability of Lipase MER to hydrolyze medium and long-chain fatty acids was weaker than that of Palatase. The use of Lipase AY 30G was accompanied by the production of some other flavor esters, which made the final hydrolysates more fragrant and may be a good choice to produce fruity cheese flavor EMC. While Lipase MER may barely contain ester activity. This study may provide a reference for the selection of incubated enzymes for specific flavor EMC.

## 1. Introduction

Cheese is widely used as a food ingredient because of its rich flavor and unique processing characteristic [1]. However, the application of natural cheese as a flavor ingredient is limited due to its low cheese flavor intensity and huge economic cost. The functions of cheese can be extended through secondary processing [2,3,4]. One of the most commonly used cheese substitutes is enzyme-modified cheese (EMC), which is treated with exogenous enzymes to increase 15–30 folds of cheese flavor intensity [5,6,7]. EMC production technologies mimic the ripening process of natural cheese, and EMC can achieve natural cheese flavor requirements with very little addition. Moreover, different flavors can be produced from different process conditions, and there are many kinds of EMCs in the foreign market. However, there is little scientific literature about the production procedures of EMC.

EMC is usually made by unmatured cheese curd with additions of emulsified salt and water. Then, exogenous enzymes play an essential role in flavor development during proteolysis and lipolysis. As proteolysis and lipolysis may vary depending on the properties of the substrate, management of a one-stage process based on the simultaneous proteolysis and lipolysis of the cheese curd is challenged [8,9,10]. A two-stage process in which proteolysis followed by lipolysis provides a possibility for easy flavor control.

Previous literature reported EMC based on commercial cheddar cheese, the authors studied the compositions, proteolytic, lipolytic, and glycolytic indices of cheddar cheese, then used a novel two-stage process to produce EMCs, and produced EMC like commercial product flavor [11,12,13]. Similar research for EMC with cheesy flavor was developed [5]. There have also been reports aimed at obtaining EMC with the flavor of ripened Turkish white cheese [14,15]. These studies provide a systematic approach for producing specific EMCs with specific cheese flavors. However, the literature is lacking studies aiming to achieve various cheese flavors and explain what enzymes can be used to achieve what effect based on one substrate. Furthermore, few studies have analyzed the effects of ripening indexes of EMC on flavor characteristics. Moreover, the impacts of the selected enzymes on the mature indicators such as extension and depth index of proteolysis of EMC have not been well understood. Thus, this research aims to explore the effect of different lipases on flavor compounds of EMC based on extensive proteolysis. Meanwhile, the contribution of different aroma compounds to flavor was also briefly explored. The results in this study will also provide a guideline for evaluating the relationship between the selected enzymes and degree of hydrolysis parameters, and even sensory properties.

## 2. Results and Discussion

### 2.1. Composition, Proteolysis, and Lipolysis

The composition, proteolytic, and lipolytic parameters of all samples are shown in Table 1. The pH decreased significantly in lipolysis which may be related to the generation of free fatty acids. After proteolysis, the value of pH 4.6-WSN/TN% increased from 2.47% to 82.60%, which was consistent with the literature reported [15]. Bas et al. (2019) hydrolyzed the slurry with Neutrase and Flavourzyme for 12 h and obtained approximately 80% pH 4.6-WSN/TN%. While this value was much higher than in previous studies (roughly 50%) which used other proteases [5,13]. It was speculated that the extent of proteolysis could be increased by using Flavourzyme. pH 4.6-WSN/TN% index reflects the bitterness level of EMC. The previous study reported that some cheeses undergo extensive proteolysis [16,17]. To our knowledge, such high proteolysis has rarely been reported for EMC production with other proteases. Therefore, Flavourzyme may be a good choice in specific EMC flavors production which requires extensive proteolysis, as the other proteases may not do the trick. Besides, proteolysis results in an increase in peptides and free amino acids. The latter contributes to the basic cheese flavor. The effect of proteolytic treatments on the mean concentration of individual FAAs (μg/g) of the control group was evaluated statistically in Table 2. Obviously, all individual FAA were elevated in the control group compared with the substrate, reflecting the enhancement of FAA caused by addition of the proteases, and this is a prerequisite step in accelerating flavor development. In this study, most of the FAAs were branched-chain amino acids. Predominant FAAs in the control group were lysine followed by leucine, threonine, valine, arginine, and histidine. A previous study about accelerating ingredient-type Cheddar cheese flavor has also pointed out the high level of leucine, histidine, lysine, and so on [18]. Lysine was abundant in cheese and can act as a flavoring agent in food applications. 2-methylbutyraldehyde and 3-methylbutyraldehyde are usually formed from isoleucine and leucine, respectively. Both aldehydes were detected in this experiment. This implies that amino acids liberated during the proteolysis are converted into volatile flavor compounds during EMC ripening.

**Figure 1:**
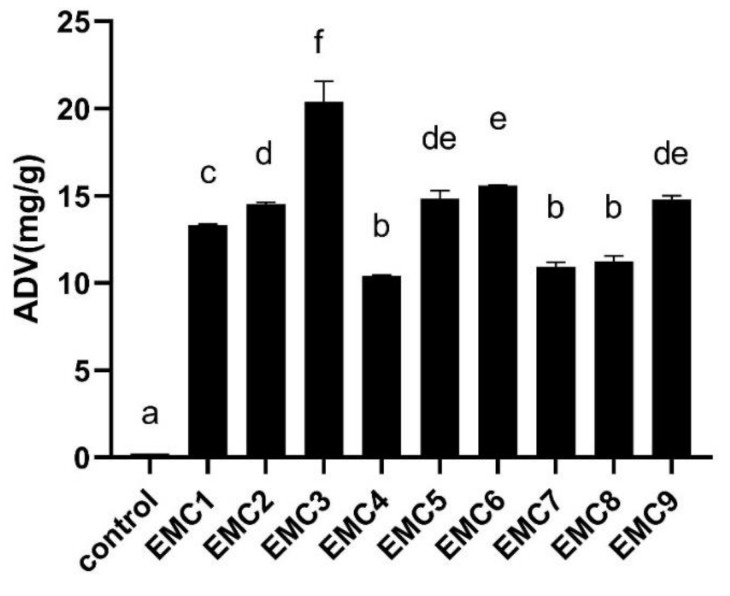
Individual acid degree values (ADV) (mg/g on a sample weight basis) of the proteolyzed product (control) and final EMCs (EMC1-EMC9). a–f: Different letters in ADV indicate significant statistical differences (Tukey’s Test, *p* < 0.05).

The level of ADV increased from 0.22% to 20.38%. Statistically significant differences (* *p* < 0.05) were found between EMC3 and other EMC products for the levels of ADV (Figure 1). The value of ADV increased with the incubation time extended by using the same lipase, which reflected the lipolysis degree. Furthermore, Table 3 mainly showed medium and long-chain fatty acids in all samples. The content of C16:0 was the highest long-chain fatty acid in the control group and EMC1-EMC9; similar results were previously reported [12]. Other predominant FFA in most EMC were C10:0, C12:0, C18:1, C14:0, and C18:0. While they contribute less to final product flavor because of their high perception thresholds. The predominance of these fatty acids mainly reflects their abundance in milk fat, but not because they are preferentially released from triglycerides [19]. In all EMCs, EMC2 had the highest total medium and long-chain FFA content, followed by EMC6, EMC1, EMC3, and EMC7. With the prolongation of hydrolysis time, the three kinds of lipases showed three different changing trends. The total content of medium and long-chain FFAs increased first and then decreased by using Palatase, and reached the highest point at about 16 h. According to the literature [8], this could be related to some FFAs converted into other substances by chemical reactions. However, in this experiment, almost no new volatile compounds were detected within 16–24 h (EMC2, EMC3), which may be due to the high peak area of acid substances in the tested samples, so that some substances with low peak area could not be detected. Moreover, in the EMCs (EMC4-EMC6) made by Lipase AY Amano 30G, the total content of medium and long-chain FFAs were gradually increased. Furthermore, the content of medium and long-chain free fatty acids of EMCs made by Lipase MER was significantly lower than that of EMCs made by Palatase, while in the analysis of the volatile compounds, the peak area of short-chain fatty acids show the opposite trend. This suggested that Palatase and Lipase MER have completely different enzymatic properties. In other words, the ability of Lipase MER to hydrolyze short-chain fatty acids was stronger than that of Palatase, while the ability of Lipase MER to hydrolyze medium and long-chain fatty acids was weaker than that of Palatase.

FFAs are precursors for a series of reactions and contribute to some volatile compounds’ formation such as esters, ketones, methyl, secondary alcohols, and lactones. In general, saturated fatty acids are higher than unsaturated fatty acids, which is beneficial to the development of a good cheese flavor. The main reason is that unsaturated fatty acids, especially polyunsaturated fatty acids, will produce various unsaturated aldehydes with strong flavor after being oxidized, leading to spoilage flavor.

### 2.2. Volatile Compounds

**Figure 2:**
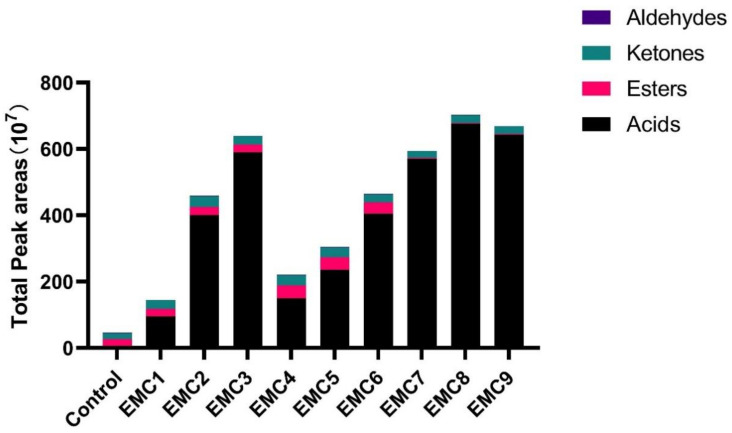
Total peak areas (10^7^) of the four volatile compounds in the control group and each EMC product.

Volatile compounds (area units, AU, ×10^7^) in proteolytic slurry and EMCs are shown in Table 4. A total of 10 compounds were detected in proteolytic products (control group): 4 esters, 4 ketones, and 2 aldehydes. Compared to the proteolytic slurry (control group), 16 new volatile compounds (7 acids, 7 esters, 2 ketones) were generated during lipolysis. Moreover, four types of fatty acids (n-butanoic acid, n-hexanoic acid, n-octanoic acid, and n-decanoic acid) from the newly formed compounds were detected in all EMC products. The total content of chemical groups of volatile compounds of EMCs increased with the incubation time extension. EMC5 had the largest variety of volatile compounds, followed by EMC4, EMC6, and EMC2. Figure 2 showed the total peak areas (10^7^) of the four kinds of volatile compounds in all samples. The total peak areas of acid compounds accounted for the largest proportion in these four kinds of volatile compounds, followed by esters and ketones compounds. Especially in EMC7-EMC9, the volatile substances consist almost entirely of acids.

Carboxylic acids were the most abundant volatile compounds isolated in the headspace of EMCs at all times of lipolysis with a percentage of 65.9–96.3% of total compounds. EMCs (EMC7–EMC9) made by Lipase MER had the highest peak areas percentage of acids. Moreover, n-butanoic acid and n-hexanoic acid were the main acid compounds. Acids accounted for 87.2% of total volatile compounds in EMC2 and EMC6, and there are very close values that support these results available in EMC studies in the literature [14]. Butanoic acid plays an important role in the flavor of many cheese types such as Cheddar, Swiss [20]. Pentanoic acid and heptanoic acid were found in EMCs prepared by Palatase and Lipase MER. Pentanoic acid was detected as nutty, grain flavor.

Compared to the proteolytic product, the total content of esters in EMC1, EMC2, and EMC3 was slightly different, while some other flavor esters are produced in EMC4, EMC5, and EMC6, especially ethyl butanoate and ethyl hexanoate. In EMC7–EMC9, the content of esters tended to decrease. That means the use of Lipase AY 30G was accompanied by the production of some other flavor esters, which made the final hydrolysates more fragrant, while Lipase MER may barely contain ester activity.

Most esters encountered in cheese are described as having sweet, fruity, and floral notes. Ethenyl acetate was detected in all products and was perceived as a fruity and sweet aroma. As far as we are concerned, this ester has not been previously isolated in EMC products, and they could provide unique and characteristic aromatic notes to the EMCs. 2-methylpropyl butanoate and pentyl butanoate were only detected in EMCs incubated by Lipase AY 30G. Ethyl butanoate and ethyl hexanoate were the main ethyl esters in EMCs by Lipase AY 30G and Palatase. Both of them contribute to fruity cheese flavor and can suppress the overall intensity of cheese [21]. Therefore, this experiment provides a basis for the production of different target flavors of EMC, for example, to produce EMC with fruity cheese flavor such as Swiss cheeses, Lipase AY 30G may be a good choice.

Ketones, especially methyl ketones (such as 2-heptanone, 2-nonanone, 2-undecanone) were found in all samples, which are known to be formed from FFA via β-oxidation reactions [8]. They are primarily known for their contribution to the aroma of surface-mold ripened and blue cheeses and they have typical odors and low perception thresholds [22]. Compared with the proteolytic products, the concentration of 3-hydroxybutanone (sour milk flavor) significantly decreased over incubation time, while higher amounts of 2-heptanone (fruity, sweet flavor) and 2-nonanone (hot milk flavor) were detected significantly in EMC1-EMC9 (* *p* < 0.05). 2-heptanone and 2-nonanone were also reported as flavor characteristics in numerous cheeses [23].

The content of the aldehydes was the lowest of the detected compound groups. In cheese, aldehydes are not the major compounds, as they are rapidly reduced to primary alcohols or even oxidized to the corresponding acids. The low level of aldehydes indicated an optimal maturation because a high concentration of aldehydes may cause off-flavors. In EMCs prepared by Palatase and Lipase MER, only 3-methylbutyraldehyde was detected.

### 2.3. Aroma Compounds

In many cases, only a small fraction of the most abundant volatile compounds may play a role in cheese flavor. Taste and aroma are very important features of cheese quality because consumers make their choice of cheeses primarily based on the characteristics of the flavor. After the substrate was incubated at 16 h by the three lipases, both the variety of volatile compounds reach its maximum, and esters and acids compounds accounted for the vast majority of the total volatile substances. Therefore, aroma characteristics and aroma intensity of volatile compounds in the headspace of EMC2, EMC5, and EMC8 are shown in Table 5. In general, the aromatic substances and their strength were improved after the hydrolysis of fat, especially esters and acids, such as ethyl butanoate, decanoic acid, octanoic acid, and hexanoic acid. Samples after lipolysis had an obvious rancid and pungent taste, especially when hydrolyzed by Palatase and Lipase MER. In EMC2, rancid, roast potato, and the pungent taste were strong, and there was also a hint of fruity flavor, while EMC5 had a more intense overall fruity flavor and less rancid smell. In EMC8, the rancid and pungent flavor were very strong, which was considered by most Chinese to be an unpleasant smell. There were still some unidentified compounds that have typical aromas at the olfactory port. An intense roast potato aroma was perceived at the olfactory port at a retention index of 909.2. Roasty odor was one of the typical aromas that was isolated at the olfactory port in the retention index of 1091. These aromas contribute to the overall flavor of EMCs.

## 3. Conclusions

The key processing techniques, chemical properties, and flavor characteristics of enzyme-modified cheese were comprehensively evaluated. The present study showed that the usage of Flavourzyme may enhance proteolysis extent, which suggested that the enzyme might be used to speed up the ripening of cheeses that need extensive proteolysis. Under certain conditions of proteolysis, the usage of Lipase AY 30G may produce some other flavor esters and make the final EMC more fragrant. Lipase MER has a stronger ability to hydrolyze short-chain fatty acids than Palatase, and Palatase has a stronger ability to hydrolyze medium and long-chain fatty acids than Lipase MER. The combination of different proteases and lipases significantly affects the final EMC flavor. This study may provide a reference for the selection of incubated enzymes for specific flavor EMC.

## 4. Materials and Methods

### 4.1. Materials

Immature cheese curd made with whole milk (Beijing Sanyuan Food Co., Ltd., Beijing, China) is used as a raw material in EMC production. Disodium hydrogen phosphate and trisodium citrate (Lianyungang Kexin Chemical Industry Co., Ltd., Jiangsu, China) were adopted as emulsifying salts. The above ingredients were blended by using a cheese melting pot (RHG-10, Institute of Food Science and Technology, CAAS, Beijing, China). Then, proteolysis and lipolysis were performed in 5 L fermenters (Shanghai Baoxing Bio-Engineering Equipment Co., Ltd., Shanghai, China). Three lipases were randomly selected according to their different substrate specificity.

The specification of proteases and lipases were presented in Table 6. Pangbo papain and Flavourzyme were obtained from Guangxi Nanning Pangbo Biological Engineering Co., Ltd. (Guangxi, China), Shanghai Branch and Novozymes (Copenhagen, Denmark), respectively. The proteinase preparations were selected based on identified differences in their specificity. Endopeptidase means randomly hydrolyzing internal peptide bonds, and exopeptidase liberated amino acids by the hydrolysis of terminal peptide bonds. Three different lipases were investigated for lipolysis, containing Palatase obtained from Novozymes (Copenhagen, Denmark), Lipase AY Amano 30G, and Lipase MER from Amano Enzyme Manufacturing (Shanghai, China), Ltd. Shanghai Branch. The latter two lipases were chosen based on differences in their hydrolytic specificity towards lipase and esterase substrates. According to the supplier, Lipase AY 30G mainly hydrolyzes the 1,2,3 positions of the glyceride, while Lipase MER hydrolyzes glyceride at 1,3 position is much greater than that at 2 positions. Besides, Palatase is a lipase that hydrolyzes dairy fats to form the free fatty acids characteristic for ripe cheeses, especially from goat’s and ewe’s milk.

### 4.2. Processing Procedure

The cheese slurry substrate (10 kg) was constituted by 4.93 kg immature cheese curd, 4.92 L deionized water, 50 g disodium hydrogen phosphate, and 100 g trisodium citrate. The ingredients were blended to a homogeneous paste and heated to 80 °C for 20 min. Subsequently, the blend was cooled to 54 °C and dispensed 4 kg into sterile containers for enzyme hydrolysis. Proteolysis was followed by lipolysis [13,15].

**Figure 3:**
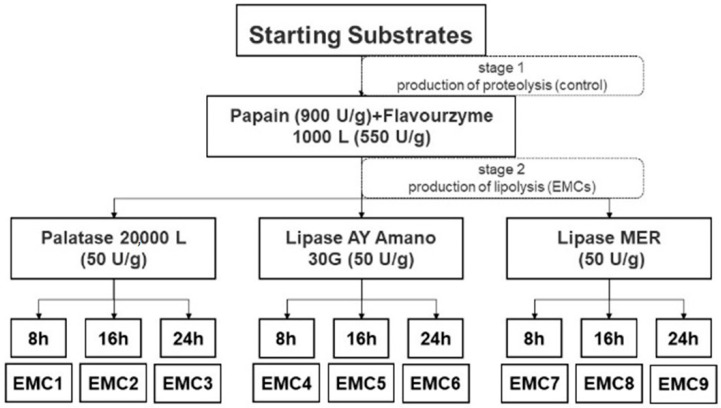
Process flow chart for the two-stage production of EMCs. Substrate: cheese slurry without enzyme hydrolyzed. Control: slurry hydrolyzed by Papain and Flavourzyme.

Pangbo papain at 900 U/g and Flavorzyme at 550 U/g were successively added for proteolysis. The incubation time was at 54 °C, with a speed of 300 rpm for 20 h. Proteolysis was terminated by heat treatment at 80 °C for 20 min. The proteolyzed slurries were quickly cooled to 50 °C and samples were frozen at −18 °C for analysis. Subsequently, single-factor experiments were performed to investigate the effects of incubation time (8 h, 16 h, 24 h) on EMC flavor with three types of commercial lipases. Briefly, Palatase 50 U/g, Lipase AY Amano 30G 50 U/g, and Lipase MER Amano 50 U/g were individually added to the proteolytic slurry. Keep lipolysis temperature at 50 °C with a speed of 300 rpm constant, no pH control, and terminate at 80 °C for 20 min. All of the enzyme concentration and incubation environments were selected according to some preliminary productions and recommendations from suppliers. Each sample was produced in triplicate and stored at −18 °C for analysis. The process flow chart for the production of EMCs is shown in Figure 3.

### 4.3. General Composition Analysis

Products were analyzed for moisture by the direct drying method (GB 5009.3-2016), NaCI by potentiometric titration (GB 5009.44-2016), protein by Kjeldahl method (GB 5009.5-2016), fat by acid hydrolysis method (GB 5009.6-2016), and pH was measured by DELTA 320 pH meter (Mettler Toledo Co., Ltd., Zurich, Switzerland). All analyses were performed in triplicate.

### 4.4. Proteolysis and Lipolysis Analysis

The contents of total N soluble in water at pH 4.6 (pH 4.6-WSN/TN%) or in 5% phosphotungstic acid (5% PTA-N/TN) were used as proteolytic index [5,15]. Nitrogen was determined by the macro-Kjeldahl method. The determination method of pH 4.6-WSN/TN% is as follows: sample was mixed with distilled water at a ratio of 1:2, stirred with magnetic force at room temperature for 5 min, heated at 50 °C for 1 h, and then cooled with 1 M HCI to adjust pH to 4.6. After centrifugation at a speed of 6000 r/min for 20 min, a certain volume of supernatant was taken for Kjeldahl determination. About 10 mL water-soluble nitrogen was taken, and 7 mL 3.95 M sulfuric acid and 3 mL 33.3% (W/V) PTA were added, mixed evenly, and placed overnight at 4 °C. Filtered by filter paper, a certain volume of the filtrate was taken for Kjeldahl determination. The levels of free amino acids (FAAs) were determined with some modification of literature [24]. The pH 4.6 WSN extracts were deproteinized by mixing equal volumes of 12% trichloroacetic acid, and allowed to stand for 10 min and then centrifuged at 14,400× *g* for 10 min. The supernatant was analyzed using an automatic amino acid analyzer (L-8900; Hitachi, Japan). All analyses were performed in triplicate. Lipolysis was analyzed by measuring the acid degree value (ADV) and free fatty acids (FFAs) of the samples. ADV was determined by hot ethanol indicator titration (GB 5009.229-2016). To determine FFA, the gas chromatographic (GC) method was performed with some modifications [25]. The sample was methyl esterified before entering the gas phase. The working conditions of GC are as follows: column was a Varian CP-Sil 88 (100 m × 0.25 mm × 0.2 μm); inlet temperature of 200 °C. The carrier gas was nitrogen with high purity (99%), held at a constant flow of 1.0 mL/min. The injector was held at 80 °C for 1 min, and the temperature was raised to 200 °C at 2 °C/min for 2 min. Then the temperature was raised to 230 °C at 5 °C/min and this temperature was held for 15 min. The flame ionization detector was operated at 300 °C; airflow was 300 mL/min, hydrogen flow was 30 mL/min; the tail blow velocity is 29 mL/min. Results were expressed as mg/100 g and all samples were analyzed in triplicate.

### 4.5. Volatile Compounds Analysis

The volatile compounds are determined by solid-phase microextraction-gas chromatography and mass spectrometry (SPME-GC/MS) [26]. Volatile compounds extraction was carried out into the vial using a 50 μm divinylbenzene/Carboxen/polydimethylsiloxane SPME fiber (Shanghai ANPEL Scientific Instrument Co., Ltd., Shanghai, China). The fibers were exposed to the headspace at 50 °C for 20 min. After extraction, compounds were directly desorbed into the 250 °C injection port. Volatile compounds were separated using a capillary column (DB-5 ms; 30 m × 250 μm ID × 0.25 μm film thickness; Agilent, Santa Clara, CA). The carrier gas was helium with a flow of 1.2 mL/min. The oven temperature was programmed initially held at 40 °C for 3 min, then the temperature was raised to 200 °C (5 °C/min, held 3 min) to a final temperature of 230 °C (10 °C/min). MS (7890A-7000B, Agilent, American) was operated in electron impact mode (mass range 40–550) with an electron impact energy of 70 eV.

### 4.6. Aroma Compounds Analysis

Gas chromatography and olfactometry (GC-O) (Sniffer9000, Brechbuhler, Switzerland) were used for aroma compounds analysis. The EMCs were extracted by the SPME method and analyzed by gas injection port. The effluent was separated by mass spectrometry and olfactory detection port (ODP), and sniffed by three well-trained sensory evaluators. Both the aroma description and intensity were recorded. The odor and timing of each compound must be consistent with the description of at least two evaluators. The perceived aroma intensity was relatively scaled as “very strong, strong, medium, weak, and very weak”. To minimize the possibility of missing potentially important aroma compounds due to human fatigue, duplicate runs were performed on all samples to confirm aroma detection. An aroma intensity was reported as “strong” if one of the two analyses detected it was “strong”. Odor-active aroma compounds were tentatively identified by mass spectrometry and confirmed by retention index and/or aroma quality. Reference samples and descriptive terms used for flavor profiling of EMC samples are shown in Table 7 [27].

### 4.7. Statistical Analysis

The differences in the volatile profiles from EMCs by different lipases at different times were analyzed using the analysis of variance (ANOVA) procedure of PASW Statistics, Version 18.0. HSD Tukey’s test was applied to compare the mean values of the volatile compounds of different samples.

## Figures and Tables

**Table 1:** General composition and hydrolysis indicator of the curd substrate, control, and EMCs (EMC1-EMC9).

Type	Moisture (%)	Protein (%) ^x^	Fat (%) ^x^	NaCI (%) ^x^	WSN (%TN) ^y^	PTA–N (%TN) ^y^	pH	ADV (mg/g)
substrate	67.50 ± 0.24 ^c^	39.40 ± 0.08 ^e^	52.00 ± 0.78 ^c^	3.02 ± 0.02 ^a^	2.47 ± 0.43 ^a^	0.35 ± 0.32 ^a^	5.89 ± 0.00 ^i^	0.056 ± 0.00 ^a^
control	66.54 ± 0.43 ^b^	36.90 ± 0.06 ^bc^	51.88 ± 0.34 ^bc^	3.12 ± 0.06 ^ab^	82.60 ± 3.00 ^bc^	16.00 ± 0.89 ^bc^	5.82 ± 0.02 ^h^	0.22 ± 0.00 ^a^
EMC1	65.78 ± 0.09 ^a^	38.45 ± 0.12 ^d^	50.24 ± 0.56 ^a^	3.08 ± 0.00 ^a^	81.58 ± 0.98 ^b^	14.88 ± 0.56 ^b^	5.42 ± 0.01 ^e^	13.31 ± 0.08 ^c^
EMC2	65.44 ± 0.16 ^a^	38.04 ± 0.09 ^d^	51.00 ± 0.23 ^b^	2.99 ± 0.05 ^a^	81.02 ± 0.78 ^b^	15.23 ± 0.09 ^b^	5.33 ± 0.01 ^c^	14.54 ± 0.08 ^d^
EMC3	66.23 ± 0.02 ^b^	36.46 ± 0.25 ^b^	50.49 ± 0.19 ^a^	3.00 ± 0.02 ^a^	79.88 ± 1.23 ^b^	14.23 ± 0.03 ^b^	5.22 ± 0.01 ^a^	20.38 ± 1.19 ^f^
EMC4	67.85 ± 0.12 ^cd^	36.78 ± 0.00 ^b^	51.25 ± 0.16 ^b^	3.14 ± 0.07 ^ab^	81.45 ± 1.00 ^b^	15.88 ± 0.14 ^b^	5.58 ± 0.01 ^g^	10.39 ± 0.08 ^b^
EMC5	66.89 ± 0.08 ^bc^	35.97 ± 0.10 ^a^	50.25 ± 0.45 ^a^	3.04 ± 0.02 ^a^	80.78 ± 0.56 ^b^	14.99 ± 0.09 ^b^	5.48 ± 0.04 ^f^	14.83 ± 0.48 ^de^
EMC6	67.70 ± 0.15 ^cd^	36.45 ± 0.04 ^b^	51.87 ± 0.33 ^bc^	3.45 ± 0.04 ^d^	80.01 ± 0.78 ^b^	14.57 ± 0.15 ^b^	5.40 ± 0.01 ^de^	15.58 ± 0.04 ^e^
EMC7	65.97 ± 0.06 ^ab^	37.88 ± 0.34 ^cd^	52.33 ± 0.78 ^c^	3.26 ± 0.06 ^c^	79.45 ± 2.45 ^b^	14.56 ± 0.01 ^b^	5.38 ± 0.01 ^d^	10.95 ± 0.24 ^b^
EMC8	66.57 ± 0.83 ^b^	37.24 ± 0.27 ^c^	51.79 ± 0.44 ^bc^	3.08 ± 0.10 ^a^	80.34 ± 1.45 ^b^	15.43 ± 0.89 ^b^	5.26 ± 0.01 ^b^	11.23 ± 0.32 ^b^
EMC9	66.89 ± 0.08 ^bc^	38.56 ± 0.01 ^d^	52.79 ± 0.28 ^cd^	3.00 ± 0.05 ^a^	80.78 ± 1.89 ^b^	14.78 ± 0.44 ^b^	5.23 ± 0.01 ^a^	14.77 ± 0.24 ^de^

^y^ TN, total nitrogen; ^x^ dry weight basis; data are expressed as (means ± SD). mg/g: on a sample weight basis. ^a–i^ different letters indicate significant statistical differences (Tukey’s Test, *p* < 0.05).

**Table 2:** Compositions and concentrations (ug/g) of free amino acids of the starting substrate and the proteolyzed product.

Amino Acids (ug/g)	Substrate	Control
Aspartic acid	1.09 ± 0.01 ^a^	93.19 ± 1.15 ^b^
Histidine	4.90 ± 0.00 ^a^	624.68 ± 0.36 ^b^
Serine	4.02 ± 0.00 ^a^	316.66 ± 0.39 ^b^
Glycine	1.56 ± 0.00 ^a^	80.38 ± 0.15 ^b^
Glutamic acid	9.47 ± 0.03 ^a^	470.67 ± 1.65 ^b^
Alanine	6.99 ± 0.03 ^a^	247.90 ± 1.00 ^b^
Tyrosine	3.67 ± 0.01 ^a^	482.02 ± 1.30 ^b^
Methionine	0.70 ± 0.00 ^a^	263.75 ± 0.53 ^b^
Lysine	17.61 ± 0.03 ^a^	1460.42 ± 2.81 ^b^
Valine	1.94 ± 0.00 ^a^	688.42 ± 0.25 ^b^
Leucine	8.58 ± 0.02 ^a^	1155.31 ± 0.09 ^b^
Threonine	9.48 ± 0.02 ^a^	972.37 ± 1.81 ^b^
Arginine	5.67 ± 0.02 ^a^	673.71 ± 2.94 ^b^
Proline	7.60 ± 0.01 ^a^	271.05 ± 0.25 ^b^
Phenylalanine	3.50 ± 0.00 ^a^	423.47 ± 0.60 ^b^
Isoleucine	0.58 ± 0.00 ^a^	281.14 ± 0.34 ^b^
Sum	87.36	8505.14
Umami amino acid/%	21.88	10.49
Sulfur-containing amino acid/%	0.8	3.1
Branched-chain amino acid/%	12.71	24.98
Hydrophobic amino acid/%	34.21	39.17
Bitter amino acid/%	53.97	71.17

μg/g on a dry weight basis./% the proportion of different types of amino acids in total amino acids. Data are expressed as mean ± SD. Note: Umami amino acids refer to glutamic acid, aspartic acid, glycine, and alanine. Sulfur-containing amino acid refers to methionine. Branched-chain amino acids contain valine, isoleucine, and leucine. Hydrophobic amino acids refer to alanine, isoleucine, leucine, valine, proline, phenylalanine, and methionine. Bitter amino acids refer to histidine, arginine, tyrosine, valine, phenylalanine, methionine, isoleucine, leucine, and lysine. ^a,b^ Different letters indicate significant statistical differences (Tukey’s Test, *p* < 0.05).

**Table 3:** Free fatty acid composition and their amounts (mg/100 g fat) in the analyzed samples.

FFA	Control	EMC1	EMC2	EMC3	EMC4	EMC5	EMC6	EMC7	EMC8	EMC9
C10:0	1.34 ± 0.06 ^a^	82.38 ± 1.61 ^c^	118.60 ± 3.98 ^f^	85.45 ± 0.82 ^cd^	56.43 ± 1.54 ^b^	84.43 ± 3.10 ^cd^	114.60 ± 5.50 ^f^	93.29 ± 2.45 ^d^	91.30 ± 1.20 ^cd^	94.10 ± 1.23 ^d^
C12:0	2.59 ± 0.12 ^a^	95.19 ± 0.91 ^de^	103.10 ± 1.68 ^e^	96.22 ± 2.03 ^de^	59.97 ± 2.46 ^b^	101.80 ± 2.91 ^e^	121.70 ± 2.80 ^f^	90.04 ± 1.60 ^cd^	82.53 ± 2.95 ^c^	91.34 ± 1.28 ^d^
C14:0	6.03 ± 0.36 ^a^	250.90 ± 4.16 ^e^	263.40 ± 4.79 ^e^	254.30 ± 1.78 ^e^	127.30 ± 2.42 ^b^	223.00 ± 3.13 ^d^	264.30 ± 2.12 ^e^	214.30 ± 5.23 ^d^	189.30 ± 5.58 ^c^	224.00 ± 3.13 ^d^
C14:1	0.75 ± 0.04 ^a^	14.37 ± 0.42 ^cd^	14.63 ± 0.69 ^cd^	11.25 ± 0.81 ^b^	13.49 ± 0.52 ^bcd^	18.45 ± 0.67 ^e^	19.62 ± 0.81 ^e^	15.54 ± 0.56 ^d^	12.70 ± 0.72 ^bc^	14.54 ± 0.60 ^cd^
C15:0	0.67 ± 0.19 ^a^	23.59 ± 1.33 ^d^	23.03 ± 0.20 ^d^	21.58 ± 1.08 ^d^	12.69 ± 0.81 ^b^	21.13 ± 1.75 ^c^	23.67 ± 1.29 ^d^	20.46 ± 1.86 ^cd^	16.85 ± 0.72 ^bcd^	19.89 ± 0.93 ^cd^
C16:0	21.50 ± 1.14 ^a^	760.60 ± 2.34 ^h^	741.40 ± 3.26 ^g^	695 ± 7.26 ^f^	422.60 ± 3.48 ^b^	675.40 ± 2.17 ^e^	755.50 ± 6.69 ^gh^	645.40 ± 6.31 ^d^	520.50 ± 2.90 ^c^	629.70 ± 4.58 ^b^
C16:1	1.52 ± 0.06 ^a^	25.00 ± 0.50 ^c^	25.53 ± 1.68 ^c^	22.35 ± 0.65 ^bc^	26.14 ± 0.39 ^c^	32.45 ± 1.10 ^d^	33.77 ± 2.16 ^d^	23.29 ± 0.71 ^bc^	18.97 ± 0.59 ^b^	23.44 ± 1.24 ^c^
C17:0	0.26 ± 0.06 ^a^	10.56 ± 1.41 ^d^	10.56 ± 0.56 ^d^	9.69 ± 0.80 ^cd^	5.65 ± 0.60 ^b^	8.65 ± 0.76 ^cd^	9.68 ± 0.64 ^cd^	9.27 ± 0.10 ^cd^	7.40 ± 0.17 ^bc^	8.97 ± 1.04 ^cd^
C18:0	7.42 ± 0.31 ^a^	242.80 ± 3.03 ^g^	237.60 ± 3.89 ^g^	213.80 ± 4.62 ^f^	85.76 ± 2.33 ^b^	123.30 ± 3.40 ^c^	141.80 ± 3.00 ^d^	220.60 ± 2.64 ^f^	172.50 ± 5.58 ^e^	209.20 ± 3.68 ^f^
C18:1 t19	1.03 ± 0.03 ^a^	7.11 ± 0.18 ^d^	6.12 ± 1.09 ^cd^	3.35 ± 0.33 ^ab^	4.52 ± 0.41 ^bc^	6.39 ± 0.35 ^cd^	4.59 ± 0.66 ^bc^	4.92 ± 0.20 ^bcd^	4.06 ± 0.27 ^bc^	5.73 ± 1.18 ^cd^
C18:1 c9	20.60 ± 0.13 ^a^	274.50 ± 2.31 ^de^	276.50 ± 7.29 ^e^	243.20 ± 3.54 ^c^	219.10 ± 1.29 ^b^	269.40 ± 6.31 ^de^	293.80 ± 1.71 ^f^	266.40 ± 3.90 ^de^	207.70 ± 4.11 ^b^	260.60 ± 2.92 ^d^
C18:2 t9 t12	0.37 ± 0.02 ^a^	2.60 ± 0.08 ^b^	2.65 ± 0.62 ^b^	2.37 ± 0.46 ^b^	3.07 ± 0.25 ^b^	3.48 ± 0.57 ^b^	3.59 ± 0.56 ^b^	2.72 ± 0.06 ^b^	2.16 ± 0.22 ^b^	2.65 ± 0.44 ^b^
C18:2 c9 c12	3.58 ± 0.11 ^a^	30.57 ± 0.99 ^cd^	31.64 ± 2.39 ^cd^	27.81 ± 2.08 ^bc^	30.17 ± 1.33 ^c^	36.16 ± 1.96 ^de^	38.74 ± 1.25 ^e^	28.82 ± 1.65 ^bc^	23.26 ± 0.90 ^b^	29.63 ± 0.38 ^c^
C20:0	0.00 ± 0.00 ^a^	2.10 ± 0.14 ^c^	2.25 ± 0.18 ^c^	1.95 ± 0.24 ^c^	0.94 ± 0.07 ^b^	0.62 ± 0.06 ^ab^	0.72 ± 0.04 ^b^	2.18 ± 0.04 ^c^	1.97 ± 0.11 ^c^	2.08 ± 0.43 ^c^
C18:3n3	0.43 ± 0.04 ^a^	3.38 ± 0.22 ^bc^	3.30 ± 0.26 ^bc^	2.75 ± 0.18 ^bc^	3.71 ± 0.67 ^bc^	4.24 ± 0.28 ^bc^	4.44 ± 0.32 ^c^	3.27 ± 0.33 ^bc^	2.52 ± 0.57 ^b^	3.18 ± 0.92 ^bc^
SFA	39.81 ± 1.29 ^a^	1468.12 ± 10.15 ^i^	1499.94 ± 5.95 ^j^	1377.99 ± 8.61 ^g^	771.34 ± 5.89 ^b^	1238.33 ± 5.41 ^d^	1431.97 ± 10.23 ^h^	1295.54 ± 5.03 ^f^	1082.35 ± 3.23 ^c^	1279.28 ± 2.34 ^e^
MUFA	23.90 ± 1.07 ^a^	320.98 ± 6.99 ^f^	322.78 ± 7.74 ^f^	280.15 ± 5.91 ^d^	263.25 ± 4.42 ^c^	326.69 ± 6.88 ^f^	351.78 ± 5.43 ^g^	310.15 ± 3.74 ^e^	243.43 ± 1.02 ^b^	304.31 ± 2.20 ^e^
PUFA	4.38 ± 0.42 ^a^	36.55 ± 1.89 ^c^	37.59 ± 2.45 ^c^	32.93 ± 1.50 ^c^	36.95 ± 0.94 ^c^	43.88 ± 3.12 ^d^	46.77 ± 2.64 ^d^	34.81 ± 2.32 ^c^	27.94 ± 0.02 ^b^	35.46 ± 0.32 ^c^
TFA	68.09 ± 1.67 ^a^	1825.65 ± 11.24 ^g^	1860.31 ± 11.20 ^h^	1691.07 ± 11.91 ^f^	1071.54 ± 6.01 ^b^	1608.90 ± 10.21 ^d^	1830.52 ± 11.64 ^g^	1640.50 ± 8.15 ^e^	1353.72 ± 4.30 ^c^	1619.05 ± 5.12 ^d^
Medium-chain (%)	5.78	9.73	11.92	10.74	10.86	11.57	12.91	11.18	12.84	11.45
Long-chain (%)	94.23	90.27	88.08	89.26	89.14	88.43	87.09	88.82	87.16	88.55

Data are expressed as (means ± SD). SFA: saturated fatty acid. MUFA: monounsaturated fatty acid. PUFA: polyunsaturated fatty acids; TFA: total medium and long-chain fatty acids. ^a–j^ Samples with different superscripts within an enzyme and incubation time means that samples differ significantly (*p* < 0.05).

**Table 4:** Volatile compounds (AU × 10^7^) were isolated from the control group and EMC products during lipolysis by three lipases) ^x^.

RI	Compounds	Control	EMC1	EMC2	EMC3	EMC4	EMC5	EMC6	EMC7	EMC8	EMC9
*Acids*											
848	Butanoic acid	0.00 ± 0.00 ^a^	43.80 ± 0.02 ^c^	151.30 ± 0.02 ^f^	161.10 ± 0.03 ^g^	43.10 ± 0.01 ^b^	75.50 ± 0.00 ^d^	149.70 ± 0.04 ^e^	205.70 ± 0.00 ^h^	209.70 ± 0.02 ^j^	209.20 ± 0.01 ^i^
864	Pentanoic acid	0.00 ± 0.00 ^a^	0.00 ± 0.00 ^a^	3.00 ± 0.01 ^c^	2.90 ± 0.00 ^b^	0.00 ± 0.00 ^a^	0.00 ± 0.00 ^a^	0.00 ± 0.00 ^a^	3.70 ± 0.01 ^d^	3.80 ± 0.01 ^e^	5.00 ± 0.05 ^f^
1030	Hexanoic acid	0.00 ± 0.00 ^a^	24.60 ± 0.00 ^b^	166.40 ± 0.02 ^e^	342.00 ± 0.13 ^h^	71.70 ± 0.02 ^c^	111.20 ± 0.02 ^d^	182.10 ± 0.06 ^f^	315.10 ± 0.00 ^g^	392.70 ± 0.00 ^j^	356.40 ± 0.02 ^i^
1106	Heptanoic acid	0.00 ± 0.00 ^a^	0.00 ± 0.00 ^a^	2.90 ± 0.00 ^e^	2.00 ± 0.02 ^c^	0.00 ± 0.00 ^a^	0.00 ± 0.00 ^a^	0.00 ± 0.00 ^a^	0.80 ± 0.01 ^b^	2.70 ± 0.00 ^d^	2.90 ± 0.01 ^e^
1192	*-*Octanoic acid	0.00 ± 0.00 ^a^	23.10 ± 0.02 ^b^	64.20 ± 0.02 ^h^	66.90 ± 0.03 ^i^	27.90 ± 0.00 ^c^	37.20 ± 0.00 ^d^	58.60 ± 0.05 ^g^	39.70 ± 0.00 ^e^	57.70 ± 0.01 ^f^	57.50 ± 0.57 ^f^
1373	Decanoic acid	0.00 ± 0.00 ^a^	3.60 ± 0.00 ^b^	12.40 ± 0.01 ^g^	14.80 ± 0.02 ^h^	6.50 ± 0.00 ^d^	10.80 ± 0.00 ^f^	14.40 ± 0.48 ^h^	5.30 ± 0.02 ^c^	9.60 ± 0.00 ^e^	11.10 ± 0.01 ^f^
1560	Dodecanoicacid	0.00 ± 0.00 ^a^	0.00 ± 0.00 ^a^	0.00 ± 0.00 ^a^	0.00 ± 0.00 ^a^	0.50 ± 0.00 ^b^	0.70 ± 0.00 ^c^	0.50 ± 0.03 ^b^	0.00 ± 0.00 ^a^	0.00 ± 0.00 ^a^	0.00 ± 0.00 ^a^
***Percentage (%) ^z^***		0.0	65.9	87.2	92.2	67.9	79.2	87.2	96.1	96.3	96.0
*Esters*											
<600	Ethenyl Acetate	4.60 ± 0.01 ^f^	3.80 ± 0.02 ^de^	3.30 ± 0.41 ^cd^	2.70 ± 0.01 ^b^	6.00 ± 0.02 ^g^	3.90 ± 0.00 ^e^	3.40 ± 0.02 ^cde^	3.00 ± 0.06 ^bc^	2.10 ± 0.01 ^a^	2.70 ± 0.01 ^b^
<600	Ethyl Acetate	4.60 ± 0.01 ^b^	0.00 ± 0.00 ^a^	0.00 ± 0.00 ^a^	0.00 ± 0.00 ^a^	0.00 ± 0.00 ^a^	0.00 ± 0.00 ^a^	0.00 ± 0.00 ^a^	0.00 ± 0.00 ^a^	0.00 ± 0.00 ^a^	0.00 ± 0.00 ^a^
724	Ethyl methoxy acetate	12.60 ± 0.02 ^b^	0.00 ± 0.00 ^a^	0.00 ± 0.00 ^a^	0.00 ± 0.00 ^a^	0.00 ± 0.00 ^a^	0.00 ± 0.00 ^a^	0.00 ± 0.00 ^a^	0.00 ± 0.00 ^a^	0.00 ± 0.00 ^a^	0.00 ± 0.00 ^a^
733	Pentyl formiate	4.70 ± 0.05 ^b^	0.00 ± 0.00 ^a^	0.00 ± 0.00 ^a^	0.00 ± 0.00 ^a^	0.00 ± 0.00 ^a^	0.00 ± 0.00 ^a^	0.00 ± 0.00 ^a^	0.00 ± 0.00 ^a^	0.00 ± 0.00 ^a^	0.00 ± 0.00 ^a^
800	Ethyl butanoate	0.00 ± 0.00 ^a^	3.80 ± 0.00 ^b^	5.10 ± 0.00 ^d^	4.70 ± 0.01 ^c^	9.50 ± 0.01 ^g^	7.70 ± 0.00 ^e^	7.90 ± 0.02 ^f^	0.00 ± 0.00 ^a^	0.00 ± 0.00 ^a^	0.00 ± 0.00 ^a^
955	2-methylpropylbutanoate	0.00 ± 0.00 ^a^	0.00 ± 0.00 ^a^	0.00 ± 0.00 ^a^	0.00 ± 0.00 ^a^	1.00 ± 0.02 ^b^	1.30 ± 0.00 ^c^	2.10 ± 0.03 ^d^	0.00 ± 0.00 ^a^	0.00 ± 0.00 ^a^	0.00 ± 0.00 ^a^
998	Ethyl hexanoate	0.00 ± 0.00 ^a^	12.50 ± 0.01 ^b^	15.30 ± 0.00 ^f^	14.70 ± 0.01 ^e^	14.50 ± 0.01 ^d^	13.20 ± 0.02 ^c^	16.50 ± 0.02 ^g^	0.00 ± 0.00 ^a^	0.00 ± 0.00 ^a^	0.00 ± 0.00 ^a^
1056	Pentyl butanoate	0.00 ± 0.00 ^a^	0.00 ± 0.00 ^a^	0.00 ± 0.00 ^a^	0.00 ± 0.00 ^a^	1.70 ± 0.00 ^c^	1.40 ± 0.01 ^b^	2.70 ± 0.00 ^d^	0.00 ± 0.00 ^a^	0.00 ± 0.00 ^a^	0.00 ± 0.00 ^a^
1065	1-methylbutylbutanoate	0.00 ± 0.00 ^a^	0.00 ± 0.00 ^a^	0.00 ± 0.00 ^a^	0.00 ± 0.00 ^a^	1.60 ± 0.00 ^c^	1.40 ± 0.00 ^b^	0.00 ± 0.00 ^a^	0.00 ± 0.00 ^a^	0.00 ± 0.00 ^a^	0.00 ± 0.00 ^a^
1195	Ethyl octanoate	0.00 ± 0.00 ^a^	0.60 ± 0.00 ^b^	1.10 ± 0.02 ^c^	1.20 ± 0.01 ^d^	4.00 ± 0.02 ^e^	7.50 ± 0.05 ^f^	0.00 ± 0.00 ^a^	0.00 ± 0.00 ^a^	0.00 ± 0.00 ^a^	0.00 ± 0.00 ^a^
1393	Ethyl decanoate	0.00 ± 0.00 ^a^	2.10 ± 0.01 ^e^	0.00 ± 0.00 ^a^	0.00 ± 0.00 ^a^	0.60 ± 0.00 ^b^	1.40 ± 0.00 ^d^	1.10 ± 0.00 ^c^	0.00 ± 0.00 ^a^	0.00 ± 0.00 ^a^	0.00 ± 0.00 ^a^
***Percentage (%) ^z^***		57.8	15.8	5.4	3.6	17.6	12.7	7.2	0.5	0.3	0.4
*Ketones*											
677	2-Pentanone	0.80 ± 0.01 ^a^	1.00 ± 0.00 ^a^	1.10 ± 0.01 ^a^	0.90 ± 0.00 ^a^	1.00 ± 0.02 ^a^	0.60 ± 0.84 ^a^	0.70 ± 0.01 ^a^	1.00 ± 0.00 ^a^	0.08 ± 0.01 ^a^	1.10 ± 0.09 ^a^
708	3-hydroxybutanone	12.60 ± 0.00 ^g^	10.70 ± 0.01 ^f^	7.80 ± 0.02 ^d^	6.00 ± 0.02 ^c^	17.00 ± 0.05 ^h^	9.30 ± 0.00 ^e^	7.30 ± 0.01 ^d^	3.50 ± 0.64 ^a^	5.00 ± 0.03 ^b^	6.10 ± 0.08 ^c^
889	2-Heptanone	3.10 ± 0.02 ^a^	8.80 ± 0.02 ^d^	12.10 ± 0.02 ^g^	9.90 ± 0.00 ^ef^	8.90 ± 0.00 ^d^	8.50 ± 0.00 ^c^	10.00 ± 0.17 ^f^	8.10 ± 0.00 ^b^	9.70 ± 0.01 ^e^	8.10 ± 0.00 ^b^
1091	2-Nonanone	1.00 ± 0.02 ^a^	6.00 ± 0.04 ^d^	9.20 ± 0.01 ^i^	9.10 ± 0.01 ^h^	4.20 ± 0.01 ^c^	4.10 ± 0.01 ^b^	6.00 ± 0.02 ^d^	7.80 ± 0.01 ^f^	7.30 ± 0.00 ^e^	7.90 ± 0.01 ^g^
1293	2-Undecanone	0.00 ± 0.00 ^a^	0.00 ± 0.00 ^a^	2.70 ± 0.01 ^b^	0.00 ± 0.00 ^a^	0.00 ± 0.00 ^a^	0.00 ± 0.00 ^a^	0.00 ± 0.00 ^a^	0.00 ± 0.00 ^a^	0.00 ± 0.00 ^a^	0.00 ± 0.00 ^a^
1496	2-Tridecanone	0.00 ± 0.00 ^a^	0.00 ± 0.00 ^a^	0.00 ± 0.00 ^a^	0.00 ± 0.00 ^a^	0.00 ± 0.00 ^a^	0.00 ± 0.00 ^a^	0.00 ± 0.00 ^a^	0.00 ± 0.00 ^a^	0.50 ± 0.00 ^c^	0.30 ± 0.00 ^b^
***Percentage (%) ^z^***		38.5	18.3	7.2	4.0	14.1	7.6	5.2	3.4	3.3	3.5
*Aldehydes*											
635	3-Methylbutanal	0.7 ± 0.00 ^c^	0.0 ± 0.00 ^a^	0.8 ± 0.00 ^d^	0.6 ± 0.00 ^b^	1.0 ± 0.01 ^e^	0.8 ± 0.00 ^d^	0.8 ± 0.01 ^d^	0.0 ± 0.00 ^a^	0.6 ± 0.00 ^b^	0.7 ± 0.00 ^c^
647	2-Methylbutanal	1.0 ± 0.00 ^d^	0.0 ± 0.00 ^a^	0.0 ± 0.00 ^a^	0.0 ± 0.00 ^a^	0.0 ± 0.00 ^a^	0.7 ± 0.00 ^b^	0.8 ± 0.00 ^c^	0.0 ± 0.00 ^a^	0.0 ± 0.00 ^a^	0.0 ± 0.00 ^a^
***Percentage (%) ^z^***		3.7	0.0	0.2	0.1	0.5	0.5	0.3	0.0	0.1	0.1

Data are expressed as (means ± SD). ^x^ Numbers are the mean area counts for three determinations on 2 g samples of control and EMCs. Standard errors for the three determinations were within 20%. ^a–j^ Different letters in the same row indicate significant statistical differences (Tukey’s Test, *p* < 0.05). RI: retention index. ^z^ Percentage (%): percentage of volatile compounds of each chemical group in each sample.

**Table 5:** Relative intensities of odor-active compounds in the headspace of the control group and EMCs made by the three lipases for 16 h.

Compound	RI	Identification Method	Odor Description	Odor Intensity			
				Control	EMC2	EMC5	EMC8
Ethenyl Acetate	<600	MS RI odor	Fruity, sweet	4	3	3	2
Ethyl Acetate	<600	MS RI	Fruity, pineapple	3	--	--	--
3-Methylbutanal	635	MS RI odor	Organic solvent	2	2	2	2
2-Methylbutanal	647	MS RI	Caramel, nutty	2	--	1	--
2-Pentanone	677	MS RI	Orange peel	2	2	1	1
3-hydroxybutanone	708	MS RI	Sour milk	3	2	3	2
Ethyl-methoxy acetate	724	MS RI	Fruity	2	--	--	--
Pentyl formiate	733	MS RI	Slightly ether	1	--	--	--
Ethyl butanoate	800	MS RI odor	Fruity, sweet	--	2	3	--
Butanoic acid	848	MS RI odor	Rancid	--	4	3	5
Pentanoic acid	864	MS RI	Nutty, grain	--	2	--	2
2-Heptanone	889	MS RI odor	Fruity, fatty	2	3	2	3
Unidentified	909	odor	Roast potato	4	4	4	4
2-methylpropylbutanoate	955	MS RI odor	Fruity	--	--	2	--
Ethyl hexanoate	998	MS RI odor	Fruit, melon, acid	--	3	3	--
Hexanoic acid	1030	MS RI odor	Pungent	--	4	4	5
Pentyl butanoate	1056	MS RI	sweet	--	--	2	--
1-methylbutylbutanoate	1065	MS RI	Pear-like, sweet	--	--	2	--
2-Nonanone	1091.3	MS RI odor	Hot milk	2	3	2	3
Unidentified	1091	odor	Roasty	2	3	2	3
Unidentified	1103	odor	earthy soil	3	3	3	3
Heptanoic acid	1106	MS RI odor	Fatty, rancid	--	2	--	3
Octanoic Acid	1192	MS RI odor	Body odor, sweat	--	3	2	3
Ethyl octanoate	1195	MS RI odor	Fruity, apple-like	--	--	3	--
2-Undecanone	1293	MS RI	Floral	--	2	--	--
Decanoic acid	1373	MS RI odor	Butter, sour fruit	--	2	2	2
Ethyl decanoate	1395	MS RI odor	Fruity	--	1	2	--
2-Tridecanone	1496	MS RI	Goaty	--	--	--	1
Unidentified	1504	odor	Milk	3	1	2	2
Dodecanoic acid	1560	MS RI	Soapy	--	--	2	--

The intensity of the compounds was rated from 1 to 5: very weak (1), weak (2), medium (3), strong (4), and very strong (5). Identified based on mass spectrometry, retention index, and odor. “--” Not detected by GC-MS and no odor.

**Table 6:** Features and specifications of proteases and lipases.

Enzymes	Origin	Optimum pH	Optimum Temperature (°C)	Enzyme Activity (U/g)
Pangbo papain	Papaya plant	6.0–7.0	50–55	77,715
Flavourzyme	*Aspergillus oryzae*	5.5–7.0	35–60	22,128
Palatase	*Aspergillus oryzae*	5–7	45–50	8065
Lipase AY Amano 30 G	*Candida rugosa*	3–8	45–55	11,474
Lipase MER	*Rhizopus oryzae*	4–6	45–55	8524

**Table 7:** Reference samples and descriptive terms used for flavor profiling of EMC samples.

Descriptive Terms	References ^1^
Salty	Sodium chloride solution (0.5% in water)
Bitter	Caffeine solution (0.08% in water)
Sour	Citric acid solution (0.08% in water)
Sweet	Sucrose solution (5% in water)
Milk	UHT milk
Butter-like	Butter
Fruity	20 ppm ethyl hexanoate (in 95% ethanol)
Organic solvent	No reference used
Nutty	No reference used
Orange peel	No reference used
Slightly ether	No reference used
Rancid	No reference used
Grain	No reference used
Roast potato	No reference used
Pungent	No reference used
Sweat	No reference used

^1^ Based on the reference with some modification [27].

## Data Availability

Data are contained within the article.
